# Surveillance and Molecular Identification of *Acanthamoeba* and *Naegleria* Species in Two Swimming Pools in Alexandria University, Egypt

**Published:** 2017

**Authors:** Ahmad Z. AL-HERRAWY, Mahmoud I. KHALIL, Soheir S. EL-SHERIF, Fatima A. E. OMAR, Wael M. LOTFY

**Affiliations:** 1. Dept. of Water Pollution Research, National Research Center, 12622 Dokki, Giza, Egypt; 2. Dept. of Zoology, Faculty of Science, Alexandria University, Alexandria, Egypt; 3. Dept. of Parasitology, Medical Research Institute, Alexandria University, Alexandria, Egypt

**Keywords:** Swimming water, *Acanthamoeba*, *Naegleria*, Identification, PCR

## Abstract

**Background:**

Swimming in contaminated water was reported to be associated with *Acanthamoeba* and *N. fowleri* human infections. The present study was carried out with the aim of isolation and identification of the different species of *Acanthamoeba* and *Naegleria* from two swimming pools in Alexandria University.

**Methods:**

Samples were collected from the swimming pools of Alexandria University Stadium and Faculty of Agriculture-Alexandria University during the period from May 2012 to April 2013.

**Results:**

Free-living amoebae were prevalent in the collected samples. Molecular characterization confirmed the identity of ten *Acanthamoeba* isolates and seven *Naegleria* isolates. *Acanthamoeba* T3, T4, T5, T11 and T15 genotypes were identified. *Acanthamoeba* T4 was the most prevalent genotype.

**Conclusion:**

The relatively high prevalence of *Acanthamoeba*, especially genotype T4, indicates the presence of a health hazard to swimmers particularly those wearing contact lenses. *Naegleria fowleri* was not found during the present study.

## Introduction

Most species of protozoa are free-living in water and soil habitats. In addition, they have been isolated from air samples (1, 2). Generally, they have little impact on human health. However, from among the many hundreds of species of free-living protozoa, only *Balamuthia mandrillaris*, *Naegleria fowleri*, *Sappinia pedata*, and some species of the genus *Acanthamoeba* are known to infect human (3, 4). Only *Acanthamoeba* spp. and *N. fowleri* are thought to have considerable public health significance (5).

*Acanthamoeba* is a genus of amoebae (Amoebozoa). They are found worldwide in freshwater, brackish water, seawater, and soil environments (6). Some species of *Acanthamoeba* can invade the human body through the cornea of the eye, broken skin, or by being inhaled (6, 7). The *Acanthamoeba* spp. incriminated in human infections can cause rare, but severe infections. They cause amoebic keratitis, granulomatous amoebic encephalitis, and disseminated granulomatous amoebic diseases (e.g., skin, sinus, and pulmonary infections) (8).

The genus *Naegleria* was originally grouped with the Amoebozoa because of having an amoeboid stage. However, it is now classified into the Excavata based on molecular phylogenetics (9). Although there are over thirty *Naegleria* spp., only *N. fowleri* is capable of infecting human (10). This species commonly inhabits warm freshwater and soil habitats. It infects people when contaminated water with *N. fowleri* enters the body through the nose and then migrates to the brain where it causes the usually fatal primary amoebic meningoencephalitis (PAM) in healthy people (11).

In addition to their role as pathogens, some free-living amoebae (FLA) may play a serious role as vehicles for transmission of some pathogens that replicate within the protozoan cytoplasm such as *Legionella pneumophila*, *Mycobacterium avium*, *Pseudomonas* spp., some *Chlamydia*-like microorganisms, fungi and viruses (12–16). Such amoebae can protect other engulfed microorganisms from the unfavorable environmental conditions including the usual methods of water disinfection (17–19).

In Egypt, *Acanthamoeba* (20, 21), and *Naegleria* (22–24) were isolated from environmental freshwater samples. Swimming in contaminated water was reported to be associated with *Acanthamoeba* and *N. fowleri* human infections (25).

The present study was carried out with the aim of isolation and identification of the different species of *Acanthamoeba* and *Naegleria* from the swimming pools of Faculty of Agriculture - Alexandria University, and Alexandria University Stadium.

## Materials and Methods

A one-year molecular survey was carried out to investigate the different species of *Acanthamoeba* and *Naegleria* from two swimming pools in Alexandria University.

Samples were collected from the swimming pools of Alexandria University Stadium and Faculty of Agriculture-Alexandria University during the period from May 2012 to April 2013. The swimming pools are of the outdoor type and are fed by the municipal drinking water network. Each month, five water samples, two liters each, were collected from the subsurface water of each swimming pool in sterile polypropylene bottles. Water samples were collected from the four corners and the middle of the swimming pool. In addition, a 10 ml water sample was collected in a small bottle for detection of the free residual chlorine. In addition, one pooled swab sample was collected from the wall corners of each swimming pool. The monthly mean number of swimmers using the pools per day and the immediate water temperature were recorded. Samples were stored in iceboxes and transported immediately to the Central Laboratory of Damanhur Drinking Water Treatment Plant in Behira Governorate.

### Cultivation of FLA

One liter of each water sample was separately filtered through nitrocellulose membrane filter (0.45 μm pore size and 47 mm diameter). After filtration, the membrane was placed face to face on the surface of non-nutrient (NN) agar medium seeded with living *E. coli* bacteria and incubated at 37°C for one week with daily microscopic examination using the inverted microscope. The second liter of each sample was processed exactly as the first one, except that the plate was incubated at 45 °C for the specific detection of only *Naegleria* (8, 26).

### Morphological identification of the isolated FLA

*Acanthamoeba*-like and *Naegleria*-like isolates were identified on the bases of both trophozoite and cyst morphology. Although the isolated putative *Acanthamoeba* were morphologically identified to the species level, the isolated putative *Naegleria* were morphologically identified only to the genus level due to the unapparent morphological criteria between species (27, 28). Trophozoites were identified by direct examination of in Page’s amoeba saline drop on a glass slide. Cysts were identified by examination of permanent stained slide preparations that were immersed in a staining jar containing Schaudinn’s fixative for 30 minutes, and then were stained with Field’s stain (29).

Cysts and trophozoites that failed to be categorized as either *Acanthamoeba*-like or *Naegleria*-like were grouped as unidentified FLA.

### Molecular characterization of the isolated FLA

The morphologically identified preserved putative isolates of *Acanthamoeba* and *Naegleria* were subjected to DNA extraction and amplified using genus-specific primers. DNA extraction was done by using phenol chloroform iso-amyl alcohol (30). The extracted DNA was amplified by using genus-specific primers for the genera *Acanthamoeba* (31), and *Naegleria* (32); and species-specific primers for *N. fowleri* (33). The sequences of the used primers and the sizes of the amplified products are presented in Table 1.

**Table 1:** Primer sets for *Acanthamoeba* spp., *Naegleria* spp. and *Naegleria fowleri*

**Product**	**Primer sequence**	**Organism**
About 180 bp* (47)	F: 5′-CCCAGATCGTTTACCGTGAA-3′ R: 5′-TAAATATTAATGCCCCCAACTATCC-3′	*Acanthamoeba* spp.
183 bp (48)	F: 5′-CAAACACCGTTATGACAGGG-3′ R: 5′-CTGGTTTCCCTCACCTTACG-3′	*Naegleria* spp.
311 bp (49)	F: 5′-GTGAAAACCTTTTTTCCATTTACA-3′ R: 5′-AAATAAAAGATTGACCATTTGAAA-3′	*Naegleria fowleri*

* Differing by a few bases depending on the genotype

PCR reaction mixture (50 μl) per sample was prepared as follows: DNA template (10 μl), Forward primer (1 μl, 100pmol/μl, Operon Biotechnologies, Germany), Reverse primer (1μl, 100pmol/μl, Operon Biotechnologies, Germany), MgCI_2_ (25mM; 2μl), Taq buffer (10X; 5 μl), Nucleotide dNTPs (10mM of each nucleotide; 1μl), Taq DNA polymerase (2.5 units; 1μl), and DEPC-treated water (28 μl).

The amplification was performed by using PCR thermal cycler (Biometra, Goettingrn Germany). The amplification program included an initial denaturation at 94 °C for 5min followed by 35 cycles; each cycle consisted of denaturation at 94 °C for 1min, annealing at 58 °C for 1min, and extension at 72 °C for 1min. The program included a final extension step at 72 °C for 10min. After agarose gel electrophoresis, the separated DNA fragments were visualized by using ethidium bromide.

The PCR products of FLA were separately purified using the QIA quick PCR purification kit (QIAGEN, Hilden, Germany). The purified PCR products and the appropriate sequencing primers for the 18S rDNA region (Table 1) were sent for sequencing at Tri-I Biotech, Inc., Taiwan, using a Mega BACE 1000 automatic sequencer (Healthcare Biosciences, Barcelona, Spain). The sequences were checked and compared using BioEdit software (Hitachi Software Engineering, Tokyo, Japan; http://www.mbio.ncsu.edu/BioEdit/bioedit.html). Molecular identification was based on sequence analysis of a part of the 18S rDNA region by comparison to the available *Acanthamoeba* and *Naegleria* DNA sequences in GenBank. Sequences generated during this study were submitted to GenBank.

## Results

During the present work, a total of 120 swimming pool water samples and 24 swimming pool wall swab samples were collected, cultivated and microscopically examined for the presence of FLA with special reference to the genera *Naegleria* and *Acanthamoeba* (Table 2)*.*

**Table 2:** Prevalence of FLA in samples of the examined swimming pools

***Naegleria*-like**		***Acanthamoeba*-like**		**Total FLA**		**No. of samples**	**Types of samples**	**Swimming pools**
**%**	**+ve**	**%**	**+ve**	**%**	**+ve**			
5	3	16.7	10	38.3	23	60	Water	Swimming pool of Faculty of Agriculture
0	0	16.7	2	41.7	5	12	Wall swap	
8.3	5	28.3	17	60	36	60	Water	Swimming pool of Alexandria University Stadium
0	0	41.7	5	83.3	10	12	Wall swap	
6.7	8	22.5	27	46.7	56	120	Water	Total
0	0	29.2	7	62.5	15	24	Wall swap	

**Fig. 1:**
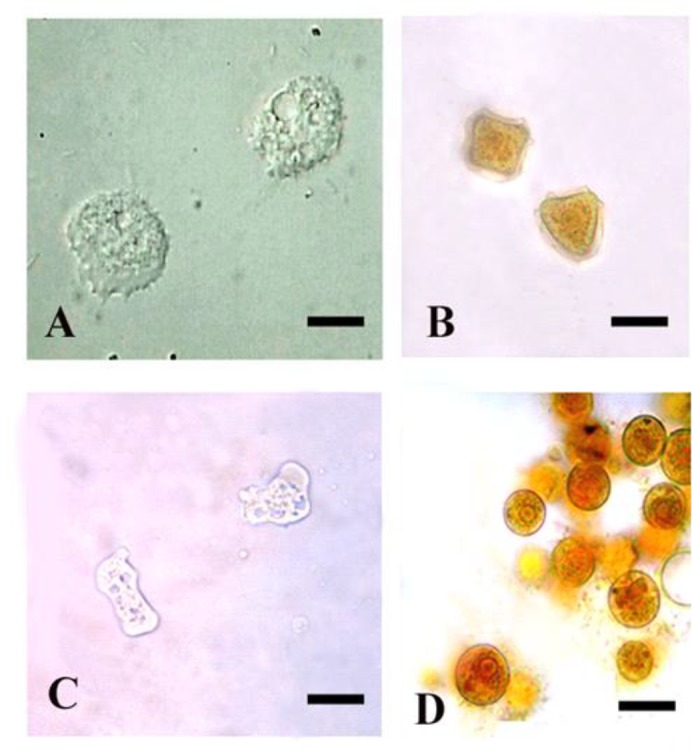
Trophozoites and cysts of *Acanthamoeba*-like (A&B) and *Naegleria*-like (C&D) FLA. Cysts are stained with Lugol’s iodine and the scale bar equals 10μm

FLA was mainly differentiated morphologically by their trophic and cystic morphotypes into three categories: *Acanthamoeba*-like (Fig. 1), *Naegleria*-like (Fig. 1), and unidentified FLA.

*Acanthamoeba*-like amoebae were present in 22.5% and 29.2% of swimming pool water samples and wall swab samples, respectively. Although, *Naegleria-*like organisms were present in 6.7% of the examined water samples, they were not present in any of the wall swab samples. Generally, occurrence of *Acanthamoeba*-like amoebae was remarkably higher than that of *Naegleria*-like amoebae in both swimming water and wall swaps (Table 2).

**Fig. 2:**
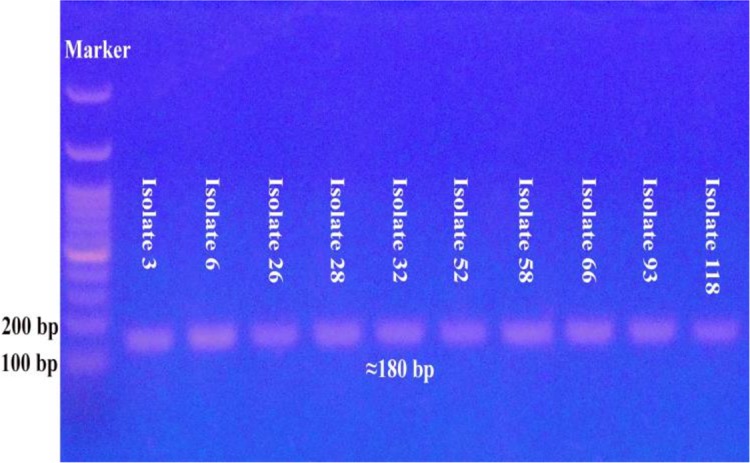
Agarose gel electrophoresis for the PCR amplified product of *Acanthamoeba* isolates DNA by using the genus-specific primers

**Fig. 3:**
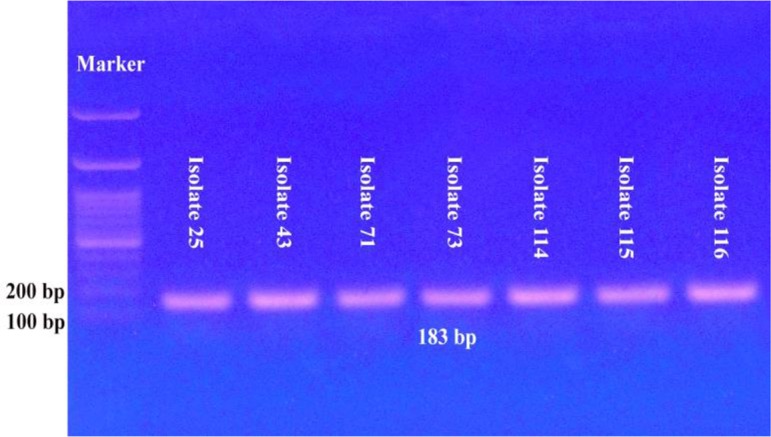
Agarose gel electrophoresis for the PCR amplified product of *Naegleria* isolates DNA by using the genus-specific primers

Molecular identification of the morphologically detected 34 *Acanthamoeba*-like isolates and the eight *Naegleria*-like isolates, using the corresponding genus-specific primers, confirmed the preliminary identification of only ten *Acanthamoeba* isolates (29.4% of the morphologically identified *Acanthamoeba*-like isolates) and seven *Naegleria* isolates (87.5% of the morphologically identified *Naegleria*-like isolates) (Fig. 2 and 3). By using the species-specific primers, none of the detected seven *Naegleria* isolates belonged to the species *N. fowleri*.

DNA sequence analysis of a part of the 18S rDNA region of the ten confirmed *Acanthamoeba* isolates (GenBank accession numbers KU312268-KU312277) revealed the presence of two isolates belonging to *Acanthamoeba* genotype T3 (isolates no. 3 and no. 66); five isolates belonging to *Acanthamoeba* genotype T4 (isolates no. 6, no. 28, no. 32, no. 93 and no. 118); one isolate belonging to *Acanthamoeba* genotype T5 (isolate no. 26); one isolate belonging to *Acanthamoeba* genotype T11 (isolate no. 52); and one isolate belonging to *Acanthamoeba* genotype T15 (isolate no. 58). Based on the results of molecular identification, *Acanthamoeba* isolates were found in the two swimming pools and they were found only during the period of May to December of the year. All the isolates, except isolate 52, were found in water samples (Table 3). On the other hand, molecular identification based on sequence analysis of a part of the 18S rDNA region of the seven *Naegleria* isolates (Gen-Bank accession numbers KU312278-KU312284) could not identify the species of any of the isolates. Based on the results of molecular identification, *Naegleria* isolates were found only in the swimming pool of Alexandria University Stadium. These isolates were only detected during four months of the year (June, July, September and December), and all the isolates were found only in water samples (Table 4).

**Table 3:** Genotypic characterization of *Acanthamoeba* isolates

**Reported sequences**	**Genotypes**	***Acanthamoeba* isolates**
100% identity with *Acanthamoeba* sp. T3 (Accession no. KJ094639; KJ094649; KJ094654; KJ094666; KJ094669; KJ476513)	T3	Isolate 3: water sample, May 2012* (Accession no. KU312268)
100% identity with *Acanthamoeba* sp. T4 (Accession no. HF930509; KP756950; KR062066)	T4	Isolate 6: water sample, May 2012* (Accession no. KU312269)
100% identity with *Acanthamoeba* sp. T5 (Accession no. AB525832; KJ652982; KJ652984; KJ652986; KM189376; KM189378)	T5	Isolate 26: water sample, Jun 2012** (Accession no. KU312270)
100% identity with *Acanthamoeba* sp. T4 (Accession no. JQ669660; JX043490; JX423579; JX423610; JX423611; KM099394; KR494236; KR780547; KR780551; KR780552; KR780553; KR780554; KR780555)	T4	Isolate 28: water sample, Jun 2012** (Accession no. KU312271)
100% identity with *Acanthamoeba* sp. T4 (Accession no. JQ669660; JX043490; JX423579; JX423610; JX423611; KM099394; KR494236; KR780547; KR780551; KR780552; KR780553; KR780554; KR780555)	T4	Isolate 32: water sample, Jul 2012* (Accession no. KU312272)
100% identity with *Acanthamoeba* sp. T11 (Accession no. KJ094683; KM189371; KM189413; KP337301; KR780557; KR780558)	T11	Isolate 52:wall swap sample, Aug 2012* (Accession no. KU312273)
100% identity with *Acanthamoeba jacobsi* (Accession no. AY262362; AY262363; AY262364; HG797013; HG797018), 99% ident with *Acanthamoeba jacobsi* (Accession no. KP233872), 99% ident with *Acanthamoeba* sp. T15 (Accession no. KJ094650; KP756945; KP756947; KP756948)	T15	Isolate 58: water sample, Aug 2012** (Accession no. KU312274)
100% identity with *Acanthamoeba* sp. T3 (Accession no. KJ094639; KJ094649; KJ094654; KJ094666; KJ094669; KJ476513)	T3	Isolate 66: water sample, Sept 2012* (Accession no. KU312275)
100% identity with *Acanthamoeba* sp. T4 (Accession no. JQ669660; JX043490; JX423579; JX423610; JX423611; KM099394; KR494236; KR780547; KR780551; KR780552; KR780553; KR780554; KR780555)	T4	Isolate 93: water sample, Nov 2012* (Accession no. KU312276)
100% identity with *Acanthamoeba* sp. T4 (Accession no. JQ669660; JX043490; JX423579; JX423610; JX423611; KM099394; KR494236; KR780547; KR780551; KR780552; KR780553; KR780554; KR780555)	T4	Isolate 118: water sample, Dec 2012** (Accession no. KU312277)

* The Swimming Pool of Faculty of Agriculture

** The Swimming Pool of Alexandria University Stadium

**Table 4:** Molecular characterization of *Naegleria* isolates by DNA sequencing

**Reported sequences with 100% identity**	***Naegleria* spp. isolates**
18S ribosomal RNA gene, partial sequence of: *Naegleria fowleri*	Isolate 25: water sample, Jun 2012** (Accession no. KU312278)
(Accession no. AF338423; AY376148; AY376149; AY376150; U80059)	Isolate 43: water sample, Jul 2012** (Accession no. KU312279)
***Naegleria lovaniensis*** (Accession no. AY376151; U80062)	Isolate 71: water sample, Sept 2012** (Accession no. KU312280)
***Naegleria* sp.** (Accession no. AF011457; DQ768717; DQ768718)	Isolate 73: water sample, Sept 2012** (Accession no. KU312281)
	Isolate 114: water sample, Dec 2012** (Accession no. KU312282)
	Isolate 115: water sample, Dec 2012** (Accession no. KU312283)
	Isolate 116: water sample, Dec 2012** (Accession no. KU312284)

* The Swimming Pool of Faculty of Agriculture

** The Swimming Pool of Alexandria University Stadium

## Discussion

Swimming pools are generally exposed bodies of water that are liable to contamination. The source of such contamination may be either environmental (like wind and rain) or by swimmers. Viruses and bacteria are usually incriminated in outbreaks associated with swimming pools. Additionally, parasitic and free-living protozoa have been reported as causative agents of illness. Protozoa can survive longer than viruses and bacteria at higher concentrations of disinfectants (34).

Results of the present study confirmed the presence of FLA in the examined samples of the two swimming pools. Morphologically, FLA were classified into three categories: *Acanthamoeba*-like, *Naegleria*-like, and unidentified FLA. The detection of FLA may indicate that the quality of the swimming water from the two examined swimming pools was not in compliance, from the parasitological point of view, with the Egyptian standards no. 418/1995. This conclusion is in accordance with that of Abd El-Salam in 2012 that assessed the water quality of five swimming pools in Alexandria (35).

Results of the molecular identification showed that only 29.4% of the morphologically identified *Acanthamoeba*-like isolates and 87.5% of the morphologically identified *Naegleria*-like isolates were confirmed to be belonging to the genera *Acanthamoeba* and *Naegleria*, respectively. This may reflect the difficulty in identification based on morphological criteria. Classical identification of FLA species was carried out based on cysts morphology, which may be inaccurate. In a previous study, molecular identification confirmed only 94.9% out of 59 morphologically identified *Acanthamoeba*-like isolates in water samples collected from swimming pools in Cairo, Egypt (20).

Molecular identification results of the present work confirmed the identity of ten *Acanthamoeba* isolates and seven *Naegleria* isolates. This may indicate that the prevalence of *Acanthamoeba* was remarkably higher than that of *Naegleria* in the water samples. This finding is in accordance with previous studies, which reported a higher prevalence of *Acanthamoeba* compared to *Naegleria* in swimming pools in Malaysia (36) and Taiwan (37). The much lower prevalence of *Naegleria* may be explained by the weaker ability of the cysts to survive in adverse conditions. For example, *N. fowleri* cysts do not survive beyond 6 months, while *Acanthamoeba* cysts can live for at least 25 years (38, 39). The relatively low prevalence of *Naegleria* in the studied swimming pools might be, also, associated with the higher sensitivity of their cysts to chlorination (40). It was suggested that the thick double-walled cysts of *Acanthamoeba* are more resistant, thus remain viable in the dry-hot areas of the platforms and in chlorinated water of the swimming pools; whereas *Naegleria* cysts are fragile and susceptible to desiccation, thus they prefer watery or moist areas for growth and proliferation (36).

According to the results of the present study, by using the species-specific primers none of the detected seven *Naegleria* isolates proved belonging to the species *N. fowleri*. This species was reported from Egyptian aquatic habitats (24). Its absence from samples collected during the present study may be explained by the fact that *N. fowleri* trophozoites and the more resistant cysts are sensitive to chlorine (40, 41). In 56 minutes, about 99.99% of *N. fowleri* cysts were killed by chlorine at a concentration of 1 mg/L added to non-turbid freshwater having a temperature of 38°C and a pH of 8.01. Turbid water requires longer disinfection times or higher concentrations of disinfectant (41). As *N. fowleri* is the only species of *Naegleria* capable of infecting and causing pathology to humans (10), its absence means that there are no hazards associated with the presence of *Naegleria* isolates in the studied swimming pools.

Results of the present study revealed that *Naegleria* was not present in the wall swabs of the swimming pools. This may indicate that, unlike *Acanthamoeba*, the biofilm microhabitat in the swimming pool wall is unsuitable for survival of *Naegleria*. Results of previous reports, which compared the distribution of *Naegleria* in sediment with water samples, are controversial (42, 43).

During the present study, the collected ten *Acanthamoeba* isolates were genotyped. Partial sequences of the 18S ribosomal RNA gene were compared to the sequences available in the GenBank. The results indicated identification of five different genotypes namely, T3, T4, T5, T11 and T15. Interestingly, among them five isolates of *Acanthamoeba* were identified as genotype T4. The five isolates were obtained from the water samples of the two swimming pools. This may indicate that *Acanthamoeba* T4 was the most prevalent genotype in the examined swimming pool samples. Previous reports by others are documenting that genotype T4 is the most common *Acanthamoeba* genotype in the environment and the most widely spread worldwide (44). It is also the main causative agent of *Acanthamoeba* keratitis (AK), granulomatous amoebic encephalitis (GAE), and capable of causing every type of *Acanthamoeba* infections (45). *Acanthamoeba* genotype T4 was isolated from skin and lung samples from HIV-infected patients (46). Moreover, T4 exhibits a significant higher binding and produces severe cytotoxicity on host cells as compared to other *Acanthamoeba* genotypes (47). Thus, the relatively high prevalence of *Acanthamoeba* genotype T4 in the examined samples presents health hazards to swimmers particularly those wearing contact lenses. During the present study, two sub clades of the genotype T4 were identified. The first sub clade was represented by isolate 6 and the other was represented by isolates 28, 32, 93 and 118. Previous studies by others are documenting that T4 is the most diverse of the genotypes of *Acanthamoeba*. It is not clear if this is simply because of the over whelming number of this genotype that has been isolated compared to other genotypes, or if this genotype is simply genetically more diverse (48).

During the present study, we identified two isolates of *Acanthamoeba* T3 genotype and one isolate of T11 genotype in the swimming pools’ samples. Genotypes T3 and T11 are closely related to T4 and have been found to be responsible for multiple cases of keratitis (49). The close genetic similarity relationship may explain why these three genotypes have all been observed in AK infections. From more than 1400 samples of all *Acanthamoeba* collected regardless of source, 84% are T3, T4 or T11 (48).

During the current study, *Acanthamoeba* T5 and T15 genotypes were isolated from water samples of the examined swimming pool. Some authors reported T5 as the second most prevalent *Acanthamoeba* genotype found (46). Genotype T5 has been isolated from numerous environmental samples. It is often associated with polluted water or sewage dump locations. Until recently with the identification of a genotype T5 isolated from AK infection, and another from a fatal infection in a heart transplant patient, genotype T5 had not been observed in an infection (50). This may indicate that genotype T5 is found in environmental locations that are unlikely to permit exposure to humans. However, T5 isolates have been found in beach and tap water surveys in close proximity to T4 (51). Therefore, at least in some cases, T5 strains are in locations where interaction with humans is possible. T15 is another *Acanthamoeba* genotype that has been found in the environment (soil and water), and caused AK infections (48).

## Conclusion

The presence of pathogenic FLA in samples collected from the two studied swimming pools indicated that the quality of water was not in compliance, from the parasitological point of view, with the Egyptian standards no. 418/1995. The relatively high prevalence of *Acanthamoeba*, especially genotype T4, in the examined samples presented health hazards to swimmers particularly those wearing contact lenses. *Naegleria fowleri* was not found during the present study. Results of the present study reflects the importance of surveillance based on molecular identification of *Acanthamoeba* and *Naegleria* species in swimming pools especially during the warm months of the year.
